# Supplementary material for: An optimized GAN method based on the Que-Attn and contrastive learning for underwater image enhancement
Source: PLoS One. 2023 Jan 6;18(1):e0279945. doi: 10.1371/journal.pone.0279945 (PMC9821782; doi:10.1371/journal.pone.0279945)
Supplement: Attachment — Submitted filename: response to reviewers.docx [file pone.0279945.s001.docx]

**Original Manuscript ID:** PONE-D-22-22875

**Original Article Title: “An Optimized GAN Method Based on the Que-Attn and Contrastive Learning for Underwater Image Enhancement**”

**To:** PLOS ONE Editor

**Re:** Response to reviewers

Dear Editor,

Thank you for allowing a resubmission of our manuscript, with an opportunity to address the reviewers’ comments.

We are uploading (a) our point-by-point response to the comments (below) (Response to Reviewers) and (b) an updated manuscript with yellow highlighting indicating changes (Revised Manuscript with Track Changes) and (c) an updated manuscript without yellow highlighting indicating changes (Manuscript).

Best regards,

Bin Zhou et al.

Journal Requirements:

**Q1. Please ensure that your manuscript meets PLOS ONE's style requirements, including those for file naming. The PLOS ONE style templates can be found at**

**https://journals.plos.org/plosone/s/file?id=wjVg/PLOSOne_formatting_sample_main_body.pdf and**

[**https://journals.plos.org/plosone/s/file?id=ba62/PLOSOne_formatting_sample_title_authors_affiliations.pdf**](https://journals.plos.org/plosone/s/file?id=ba62/PLOSOne_formatting_sample_title_authors_affiliations.pdf)

**A1:** We have checked and modified our manuscript according to journal style requirements.

**Q2. Thank you for stating the following financial disclosure:**

**"YES - Specify the role(s) played."**

**At this time, please address the following queries:**

**a)        Please clarify the sources of funding (financial or material support) for your study. List the grants or organizations that supported your study, including funding received from your institution.**

**b)        State what role the funders took in the study. If the funders had no role in your study, please state: “The funders had no role in study design, data collection and analysis, decision to publish, or preparation of the manuscript.”**

**c)        If any authors received a salary from any of your funders, please state which authors and which funders.**

**d)        If you did not receive any funding for this study, please state: “The authors received no specific funding for this work.”**

**Please include your amended statements within your cover letter; we will change the online submission form on your behalf.**

**A2：**We have declared and modified as follows, meanwhile, we have complemented in cover letter.

**Funding:** This work was supported by the Natural Science Foundation of Shandong Province under Grant ZR2021MF031 and ZR2020MF147. The funders had no role in study design, data collection and analysis, decision to publish, or preparation of the manuscript.

Competing interests: The authors have declared that no competing interests exist.

**Q3. Thank you for stating the following in the Acknowledgments Section of your manuscript:**

**“This work was supported by the Natural Science Foundation of Shandong Province under Grant No. ZR2021MF031 and No. ZR2020MF147”**

**We note that you have provided additional information within the Acknowledgements Section that is not currently declared in your Funding Statement. Please note that funding information should not appear in the Acknowledgments section or other areas of your manuscript. We will only publish funding information present in the Funding Statement section of the online submission form.**

**Please remove any funding-related text from the manuscript and let us know how you would like to update your Funding Statement. Currently, your Funding Statement reads as follows:**

**“YES - Specify the role(s) played.”**

**Please include your amended statements within your cover letter; we will change the online submission form on your behalf.**

**A3:** We have removed the funding-related text from the Acknowledgements section to the Funding Statement section and deleted the acknowledgements section. The funding information is described as follows and must be published in the Funding Statement section of the online submission form.

**Funding:** This work was supported by the Natural Science Foundation of Shandong Province under Grant ZR2021MF031 and ZR2020MF147. The funders had no role in study design, data collection and analysis, decision to publish, or preparation of the manuscript.

We have declared and complemented the Author Contributions section in manuscript as follows, meanwhile, we have complemented in cover letter.

**Author Contributions**

**Conceptualization:** Bin Zhou, Zeru Lan.

**Data curation:** Zeru Lan, Weiwei Zhao.

**Formal analysis:** Bin Zhou, Shaoqing Wang.

**Investigation:** Weiwei Zhao.

**Methodology:** Zeru Lan, Bin Zhou.

**Project administration:** Shaoqing Wang.

**Resources:** Bin Zhou, Shaoqing Wang.

**Software:** Zeru Lan, Weiwei Zhao.

**Supervision:** Shaoqing Wang, Weiwei Zhao.

**Validation:** Weiwei Zhao.

**Visualization:** Zeru Lan, Weiwei Zhao.

**Writing – original draft:** Zeru Lan, Bin Zhou.

**Writing – review & editing:** Zeru Lan, Bin Zhou.

**Q4. In your Data Availability statement, you have not specified where the minimal data set underlying the results described in your manuscript can be found. PLOS defines a study's minimal data set as the underlying data used to reach the conclusions drawn in the manuscript and any additional data required to replicate the reported study findings in their entirety. All PLOS journals require that the minimal data set be made fully available. For more information about our data policy, please see http://journals.plos.org/plosone/s/data-availability.**

**Upon re-submitting your revised manuscript, please upload your study’s minimal underlying data set as either Supporting Information files or to a stable, public repository and include the relevant URLs, DOIs, or accession numbers within your revised cover letter. For a list of acceptable repositories, please see http://journals.plos.org/plosone/s/data-availability#loc-recommended-repositories. Any potentially identifying patient information must be fully anonymized.**

**Important: If there are ethical or legal restrictions to sharing your data publicly, please explain these restrictions in detail. Please see our guidelines for more information on what we consider unacceptable restrictions to publicly sharing data: http://journals.plos.org/plosone/s/data-availability#loc-unacceptable-data-access-restrictions. Note that it is not acceptable for the authors to be the sole named individuals responsible for ensuring data access.**

**We will update your Data Availability statement to reflect the information you provide in your cover letter.**

**A4：**We have declared and complemented **Data Availability Statement** as follows, meanwhile, we have complemented in cover letter.

Data Availability Statement: Article data comes from public data sets. **UIEB** is available from URL: <https://li-chongyi.github.io/proj_benchmark.html> DOI:10.1109/TIP.2019.2955241. **HICRD** is available from URL: <https://github.com/JunlinHan/CWR> DOI:10.3390/rs14174297. **EUVP** is available from URL: <https://irvlab.cs.umn.edu/resources/euvp-dataset> DOI:10.3390/rs14174297.

**Q5.** We note that you have stated that you will provide repository information for your data at acceptance. Should your manuscript be accepted for publication, we will hold it until you provide the relevant accession numbers or DOIs necessary to access your data. If you wish to make changes to your Data Availability statement, please describe these changes in your cover letter and we will update your Data Availability statement to reflect the information you provide.

**A5：**We have described Data Availability Statement as the above mentioned. Data Availability Statement has no change, but we have complemented in cover letter again.

**Q6. We note that Figure 2 includes an image of a participant in the study.**

**As per the PLOS ONE policy (http://journals.plos.org/plosone/s/submission-guidelines#loc-human-subjects-research) on papers that include identifying, or potentially identifying, information, the individual(s) or parent(s)/guardian(s) must be informed of the terms of the PLOS open-access (CC-BY) license and provide specific permission for publication of these details under the terms of this license. Please download the Consent Form for Publication in a PLOS Journal (http://journals.plos.org/plosone/s/file?id=8ce6/plos-consent-form-english.pdf). The signed consent form should not be submitted with the manuscript, but should be securely filed in the individual's case notes. Please amend the methods section and ethics statement of the manuscript to explicitly state that the patient/participant has provided consent for publication: “The individual in this manuscript has given written informed consent (as outlined in PLOS consent form) to publish these case details”.**

**If you are unable to obtain consent from the subject of the photograph, you will need to remove the figure and any other textual identifying information or case descriptions for this individual.**

**A6:** Two pictures in Figure 2 come from the U45 public data set ([URL: https://github.com/IPNUISTlegal/underwater-test-dataset-U45-](URL:%20https://github.com/IPNUISTlegal/underwater-test-dataset-U45-) DOI:10.48550/arXiv.1906.06819). We have selected the two typical pictures from the U45 public data set and performed a RGB histogram comparison result, thus highlighted the effectiveness of our proposed method.

**Reviewer's Responses to Questions**

**Q2.** Has the statistical analysis been performed appropriately and rigorously?

Reviewer #1: Yes

Reviewer #2: No

**A2:** We have employed three common underwater image enhancement quality evaluation indicators and compared with the three quality evaluation indicator results derived from the proposed methods of other literatures. The comparision results show our proposed method can improve the three quality evaluation indicator results more than other methods. Thus our proposed method is with reasonable statistics, and plenty of experiments can further verify the reasonable statistics.

**Q4.** Is the manuscript presented in an intelligible fashion and written in standard English?

PLOS ONE does not copyedit accepted manuscripts, so the language in submitted articles must be clear, correct, and unambiguous. Any typographical or grammatical errors should be corrected at revision, so please note any specific errors here.

Reviewer #1: No

Reviewer #2: Yes

**A4:** Through carefully check, we have corrected some inappropriate description and typographical or grammatical errors.

**Review Comments to the Author**

**Reviewer #1**

**Q1: In a large number of places the paper talks about contrast learning rather than** **contrastive learning**

**A1:** There are some mistakes in our spelling, and we have corrected them all.

The title: Comparative Learning is changed to contrastive learning.

Line25: the contrast method is changed to the contrastive method.

Line28: contrast loss is changed to contrastive loss.

Line30: contrast learning is changed to contrastive learning.

Line35: contrast learning is changed to contrastive learning.

Line117: contrast loss is changed to contrastive loss.

Line129: contrast loss is changed to contrastive loss.

Line173: contrast learning is changed to contrastive learning.

Line178: contrast loss is changed to contrastive loss.

Line190: contrast loss is changed to contrastive loss.

Line196: contrast loss is changed to contrastive loss.

Line197: contrast loss is changed to contrastive loss.

Fig3: contrast loss is changed to contrastive loss.

Fig4: contrast loss is changed to contrastive loss.

Line212: “by the loss of contrastive” is changed to “by the contrastive loss”.

Line239: contrast estimation framework is changed to contrastive estimation framework.

Line241: contrast learning is changed to contrastive learning.

Table4: comparison loss is changed to contrastive loss.

**Q2: Line 172 refers to Fig 3-2. There is no such figure.**

**A2:** Sorry, we forgot to delete this sentence (i.e. The background theory is shown in Figure 3-2.) at that time, but now it has been deleted.

**Q3: Fig 3 has missing labels for** **G(I_X), I_X, I_Y,** **F_X,** **F_Y,** **A_g and** **A_{QA} which makes it hard to understand**

**A3:** Fig 3 has modified as follows.

**Materials and methods** section has modified as follows. So several mentioned labels in this question are easy to understand.

**Q4****: Fig 4 also missing labels, e.g F_X and F_Y**

**A4:** Fig 4 has modified as follows. Several mentioned labels in this question are easy to understand.

**Q5: In the caption of Fig 4 it uses "mg" presumably for "A_g"**

**A5:** “mg” should be changed to Ag, and we have corrected the problem.

**Q6:- Line 183: "Our another" should be "Our other"**

**A6:** We have corrected the problem.

**Q7:** - Line 194: The sentence ending "not important to ." seems unfinished

**A7:** We have corrected the problem**.**

**Q8:** - Line 250: "We use and a" should be "We use a"

**A8:** We have corrected the problem**.**

**Reviewer #2**

**Q1: Line 92 « Li et al [21] », Reference 21 is not authored by Li et al. Please correct.**

**A1:** Thanks. It has been corrected.

**Q2: Lines 96-98: It is said that “training on real data can produce visually satisfactory results”, but also that “they often produce unreal results because the reference image is not the actual ground truth” How real data could not be actual ground truth ?**

**A2:** It has been corrected and described in detail as follows:

In a word, the restoration eﬀect of the above methods on color and texture details is still unsatisfactory, and may produce unrealistic results. Luckily, we have achieved positive results by combining contrastive learning with attention mechanism.

**Q3: Line 172 “The background theory is shown in Figure 3-2”.There is no Figure 3-2. Please correct**

**A3:** We forgot to delete this sentence at that time, but now it has been deleted.

**Q4: Fig.2 is not documented in the core of the manuscript. We don’t know exactly what the purpose of this figure is. Please specify where the original underwater images and the restored images are.**

**A4:** We have corrected and supplemented as follows:

**Fig 2.** Comparison of RGB tricolor histograms for underwater image and underwater restoration image. Of which, the lower left figure is the input image, the upper left figure is the recovered underwater image, and the bottom right image is the input underwater image, the top right image is the recovered underwater image.

**Q5: Qualitative results on the HICRD test dataset (fig. 5) and Quantitative results are given for EUVP dataset (table 1). It would be interesting to have the two evaluations for the same dataset to compare the qualitative and quantitative results.**

**A5:** Table 1 reflects **Qualitative** and **Quantitative results** on the **EUVP** dataset, Table 2 reflects **Qualitative** and **Quantitative results** on the **UIEB** dataset, Table 3 reflects **Qualitative** and **Quantitative results** on the **HICRD** dataset, and Fig.5 reflects **Qualitative** and **Quantitative result visualization** from our proposed method and existing methods on the **HICRD** dataset. There are error descriptions relative to Table 2 and Table 3 in original manuscript, we have corrected.

**Q6: In figure 5, the authors claim the superiority of their method compared to traditional restoration methods. But the CUT, CycleGAN and CWR methods are visually sometimes more satisfying than the proposed method.**

**A6:** Table 3 reflects **Qualitative** and **Quantitative results** on the **HICRD** dataset as follows, and Fig.5 reflects **Qualitative** and **Quantitative result visualization** from our proposed method and existing methods on the **HICRD** dataset. Through quantitative and qualitative results in table 3, we can know our method has better effect than other methods, but degree of ascension is not obvious, so the visual difference on figure 5 is not obvious, but the visual effect is not inferior to other methods. In this paper, we verify the effectiveness of the proposed method by a large number of experiments, meanwhile display the results visually.

**Q7: Tables1, 2, 3, the experimental data,(EUVP, HICRD, UIEB) are presented as “synthetic underwater images”, whereas in Datasets paragraph (p.9/15), they are not.**

**A7:** We have described the dataset in more detail as follows:

**UIEB** The UIEB dataset [20] is **a composite dataset** that consists of 890 paired images, with another 60 challenging images to be restored without ground-truth values. Among them, we select a random subset of 800 images as the training set, and the remaining 90 images as the test set. The sizes of the images are 860 *×* 590.

**HICRD** HICRD [35] is **a composite dataset** that contains 9*,*676 original underwater images and 2*,*000 restored reference images. It contains two subsets, unpaired HICRD and paired HICRD. It also contains measured parameters such as diﬀuse attenuation coefficients and camera sensor responses.

**EUVP** [36] This dataset is **a composite dataset** that is collected by various cameras, such as GoPros, micro-light USB, and other cameras during ocean exploration under varying visibility conditions. It includes 5*,*550 paired test data, 3*,*200 pairs of unpaired test data, and 515 pairs of test datasets. The resolution of the images is 256 *×* 256. We use 3*,*200 unpaired data to train our network.

**Q8: The problem of time efficiency is mentioned in page 10 (“speed and memory” paragraph) but real time processing is not tackled, it is pivotal for robots’ autonomous operations. Thus, it is essential to develop a real-time and adaptive method for underwater visual restoration. What is the real-time efficiency of the proposed method and how could it be improved.**

**A8:** Our memory usage accounts for about 8G of video memory. High resolution images can process about 10 images a second, and low resolution images can process about 20 images a second. Different test sets have different processing speeds. The real-time efficiency of our method can be improved by enhancing computer hardware configuration.

**Q9:** **To provide complete information, the legends of the tables of results and the figures of results must specify the data used for learning and the test data.**

**A9:** We have checked and modified the result description of the tables and the figures. The test datasets we used are all standard datasets, and the results obtained are also compared with previous articles.

**Q10: Table 4 should be more clearly described. What is the difference between A (Ordinary GAN Network) et C (GAN Network) ?**

**A10:** We have introduced each part in more detail as follows:

**Table 4.** Quantitative results on synthetic underwater images in terms of SSIM, PSNR, and UIQM. A represents the use of ordinary GAN network, B represents the use of GAN network and contrastive loss, and C represents the use of GAN network, query attention and contrastive loss.

**Q11:** **The proposed attention-querying mechanism preserves the simple design of CUT and does not add any model parameters. However, in reference 41 (Park T, Efros AA, Zhang R, Zhu JY., European conference on computer vision. Springer; 2020. p. 319–345), the authors show results (see Fig. 3 in the cited article) with two failure cases. In the first case, they were unable to identify the unfamiliar pose of the horse and instead add texture to the background. In the second, the method added a tongue to the dog that does not exist. Have you been faced with such failure situations. If not, could you explain why.**

**A11:** We did not encounter this situation during the test. It should be that we mainly restored the color. The CUT article is mainly about style migration. The main direction is different, so we did not encounter this situation.
